# The wind changed direction and the big river still flows: from EUPHRATES to TIGRIS

**DOI:** 10.1186/s40560-019-0386-0

**Published:** 2019-05-16

**Authors:** Toshiaki Iba, David J. Klein

**Affiliations:** 1 Department of Emergency and Disaster Medicine, Juntendo University Graduate School of Medicine, 2-1-1 Hongo Bunkyo-ku, Tokyo, 113-8421 Japan; 2 Department of Critical Care, St. Michael’s Hospital, University of Toronto, Toronto, Canada

**Keywords:** Polymyxin B-immobilized fiber column, Sepsis, Septic shock, Randomized controlled trial, Endotoxin activity assay

## Abstract

The overall result of the randomized controlled double-blinded trial for polymyxin B-immobilized fiber column on septic shock (EUPHRATES trial) was disappointing. However, post hoc analysis showed benefits for patients with certain Endotoxin Activity Assay (EAA) levels. Thus, the study will be repeated, and the data will be added to the former trial. Using a precision medicine approach, eligibility criteria have been modified in TIGRIS to include patients with MODS score > 9 and EAA levels between 0.60 and 0.89. We are currently feeling the change in the wind as the rivers continue to flow towards PMX therapy for endotoxemic septic shock.

## Main text

Hemoperfusion using a polymyxin B-immobilized (PMX) fiber column (Toraymyxin™) for septic shock was initiated in the 1980s. Subsequently, its use in endotoxemic septic shock was covered by insurance in Japan in1994, and the usefulness has been extensively studied in the world [1, 2]. However, since the efficacy has not been conclusive [3], a randomized controlled trial (RCT) ‘Evaluating the Use of Polymyxin B Hemoperfusion in a Randomized controlled trial of Adults Treated for Endotoxemia and Septic shock (EUPHRATES)’ was conducted in North America [4]. EUPHRATES was a multi-centered, sham circuit-controlled and blinded trial. Patients with refractory shock and endotoxin activity assay (EAA) levels ≥ 0.6 were randomized to treatment with or without PMX hemoperfusion. An amendment to the protocol was made after the interim analysis that suggested lack of effect in the patients with Multiple Organ Dysfunction Score (MODS) of 9 or less, and those patients were excluded thereafter. The results of EUPHRATES were as follows: in 294 enrolled patients with MODS > 9, the 28-day mortality was 44.5% in the treatment group, whereas it was 43.9% in the control group. These results were quite disappointing, and we worried that a PMX study would never be repeated.

Following the first report, data from a post hoc analysis was released, and the beneficial impact of PMX in the 194 patients with refractory septic shock and EAA levels between 0.60 and 0.89 was demonstrated [5]. The risk difference was 10.7% (26.1% in the treatment group versus 36.8% in the control group), and the odds ratio was 0.52 (95%CI, 0.27–0.99, *P* = 0.047) for improvement in mortality. In addition, the PMX group demonstrated a greater increase in mean arterial pressure and improvements in ventilator-free days. Indeed, the improvement in cardiovascular dysfunction is the principal mechanism, and the result was consistent with that of other reports [1]. The reason why PMX-DHP could not show the efficacy in the patients with EAA levels of 0.9 or more is still unclear. Those patients may require the form of a greater number of cartridges, larger cartridges, or longer treatment.

We not only agree that the primary result is essential, but it is also understandable that there is a limit to the therapy’s ability to handle extreme pre-treatment burdens of endotoxin level. Romaschin et al. [6] had demonstrated that the performance of PMX was optimal in vitro only when the endotoxin level was within the specified range of EAA 0.60 to 0.89. Thus, we think this post hoc study has at least raised an important and testable hypothesis. However, the recent decision of the Food and Drug Administration (FDA) was surprising. It was announced that TIGRIS trial, a second phase III trial of PMX, has been approved as an amendment to the EUPHRATES protocol, and the new data will be added to EUPHRATES trial in a Bayesian approach. This means that the patients will be enrolled in the TIGRIS trial using the same eligibility criteria as those that showed a clinically significant mortality benefit in the EUPHRATES trial. The number of patients to be enrolled is expected to be 150, and the patients will be randomized to 2:1 fashion for treatment versus control arm (https://globenewswire.com/news-release/2019/02/19/1734223/0/en/Spectral-Announces-Approval-of-Tigris-Trial-by-the-US-FDA.html). Furthermore, the TIGRIS trial will be run exclusively in the hospitals experienced in using the PMX cartridge and who demonstrated a good enrollment rate in the former trial. The willingness of the FDA to allow this approach does appear to be surprising albeit overdue response to the lack of any available septic shock treatments on the market.

Then, what has led to the wind to change directions? Firstly, we have realized the limitations of our modalities for the sepsis treatment and the importance of precision medicine [7]. Treatment like endotoxin adsorption is only effective for the specific patients who show the particular range of circulating endotoxin level [8]. A single approach cannot be applied to all patients, and old clinical sepsis definitions may not be helpful in finding biologically similar patients to treat. As is known, sepsis is a quite heterogeneous category, and personalized medicine will enable us to choose the appropriate candidates. We should pay more attention to the patient selection and intensively conduct trials in a specifically selected group that are most likely to respond to the intervention if we are to be more successful in the future.

Undoubtedly, septic shock is one of the toughest unconquered enemies of human health. While, the progress in the development of adjunctive therapy for sepsis is still sparse, at this moment, we emphasize again that it is too early to give up the hemoperfusion with PMX and further study that catches the wind is warranted [9]. Though EEA is not popular in Japan, this assay will become popular in the world once TIGRIS succeeds. We must keep following the rivers to reach a successful end result.

## Abbreviations

CI: Confidence interval
EAA: Endotoxin activity assay
FDA: Food and Drug Administration
MODS: Multiple Organ Dysfunction Score
PMX: Polymyxin B-immobilized fiber column
RCT: Randomized controlled trial
